# Supplementary figures and images for: High-resolution, genome-wide mapping of positive supercoiling in chromosomes
Source: eLife. 2021 Jul 19;10:e67236. doi: 10.7554/eLife.67236 (PMC8360656; doi:10.7554/eLife.67236)

$\infty$  supercoiled  
 $\nabla$  nicked  
 $\circ$  relaxed

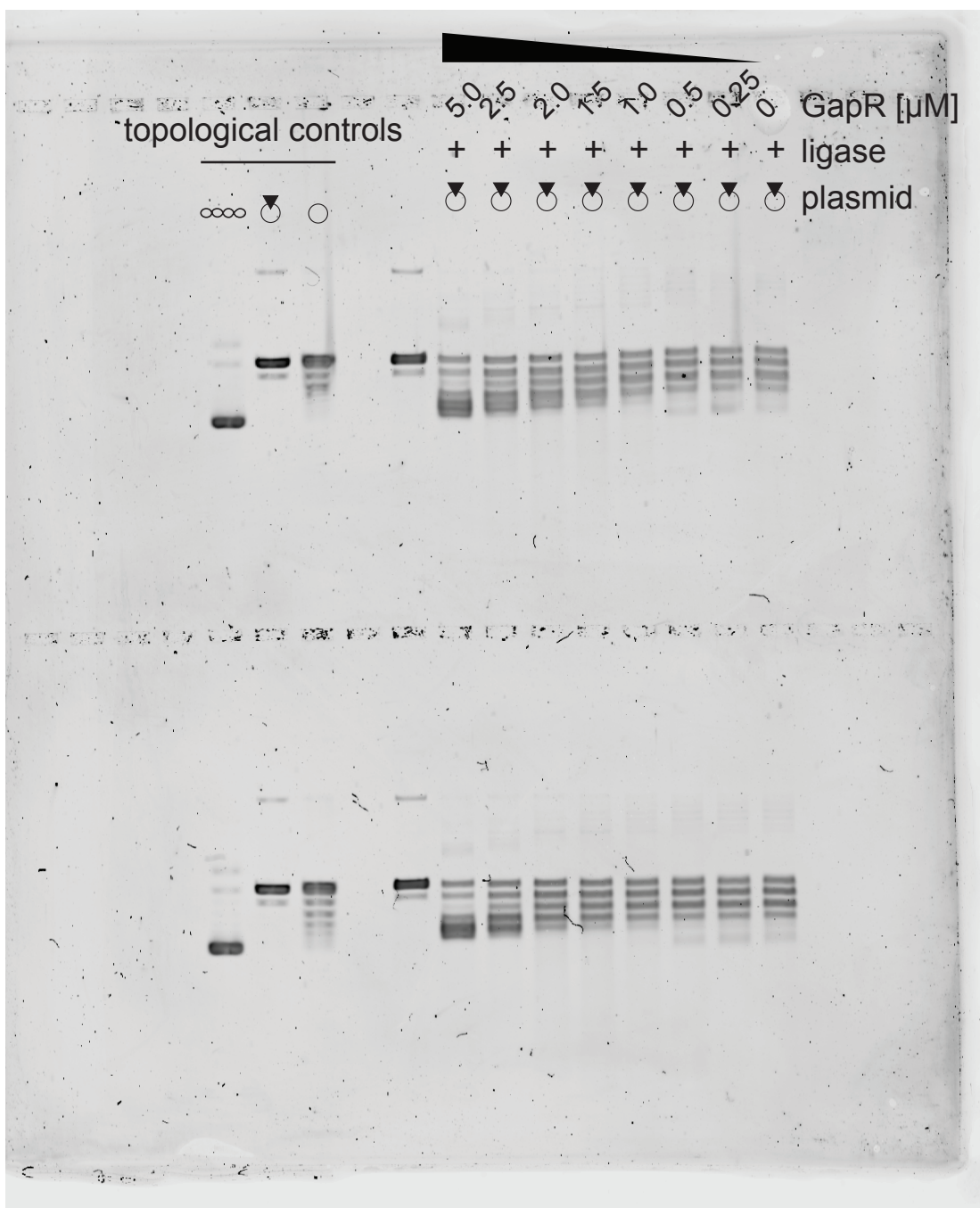

Supplement: Figure 1—source data 1. [file elife-67236-fig1-data1.zip › Figure 1A-source data 1_labeled.pdf]

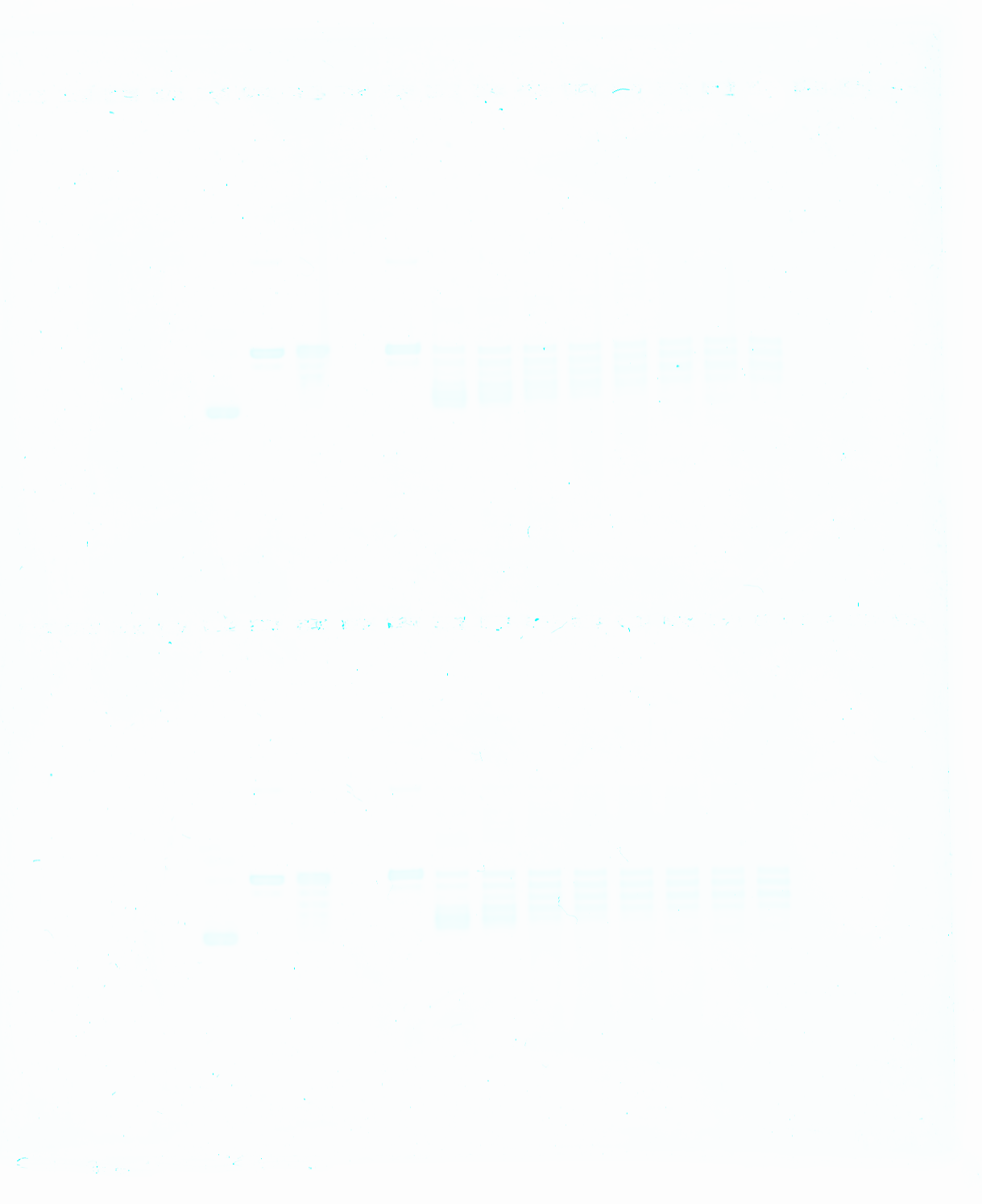

Supplement: Figure 1—source data 1. [file elife-67236-fig1-data1.zip › Figure 1A-source data 1.tif]

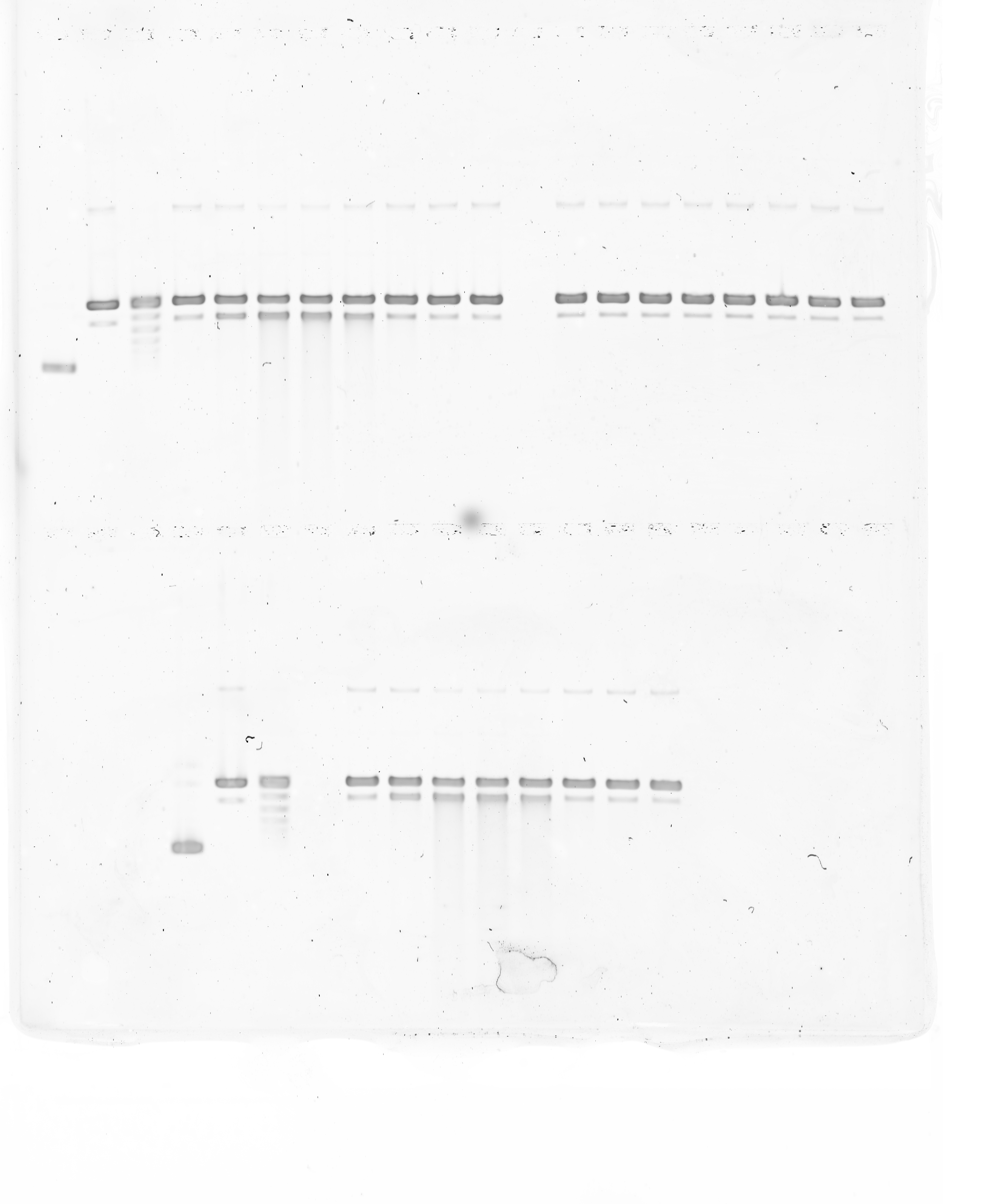

Supplement: Figure 1—figure supplement 1—source data 1. [file elife-67236-fig1-figsupp1-data1.zip › Figure 1-figure supplement 1A-source data 1.tif]

$\infty$  supercoiled  
 $\nabla$  nicked  
 $\circ$  relaxed

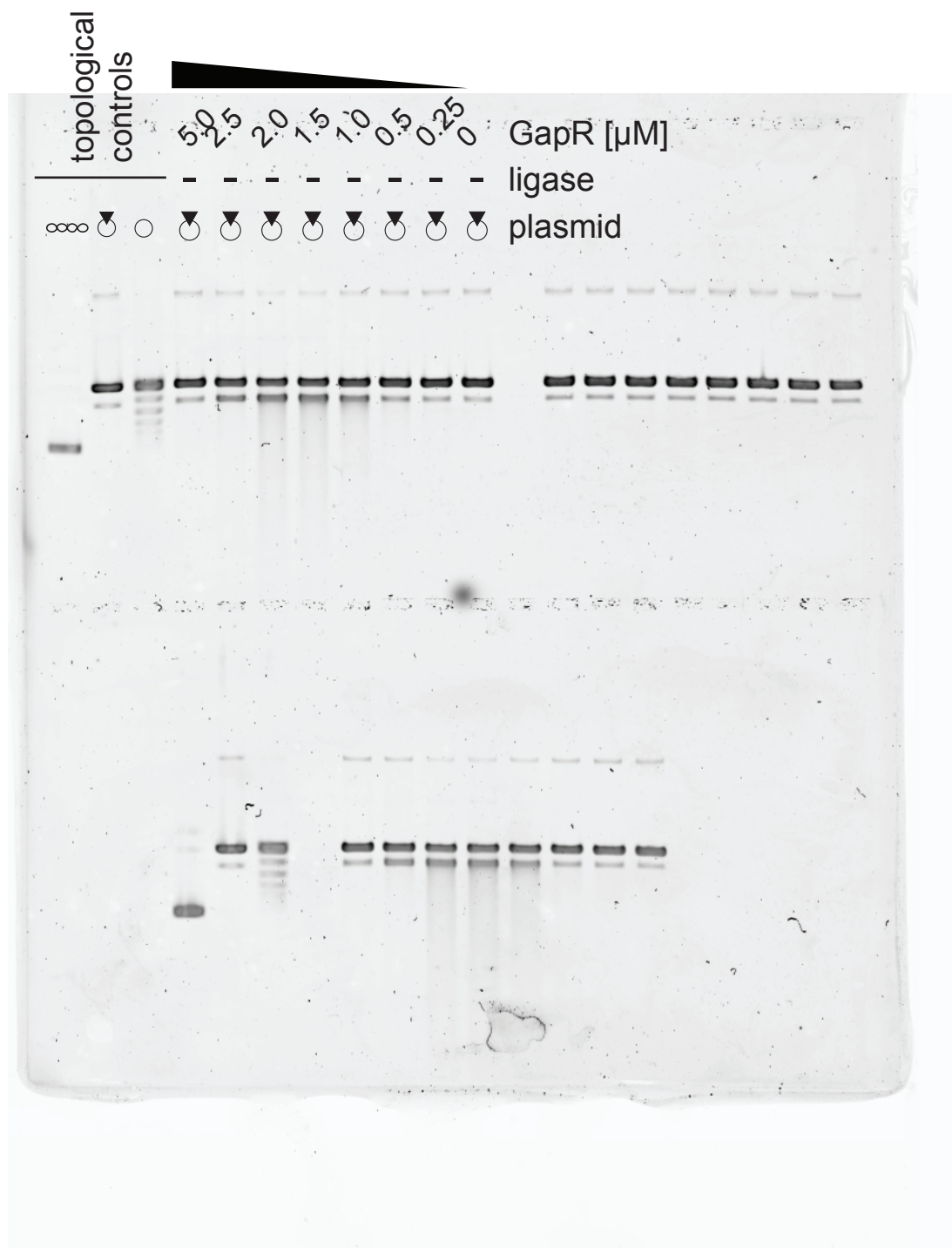

Supplement: Figure 1—figure supplement 1—source data 1. [file elife-67236-fig1-figsupp1-data1.zip › Figure 1-figure supplement 1A-source data 1_labeled.pdf]

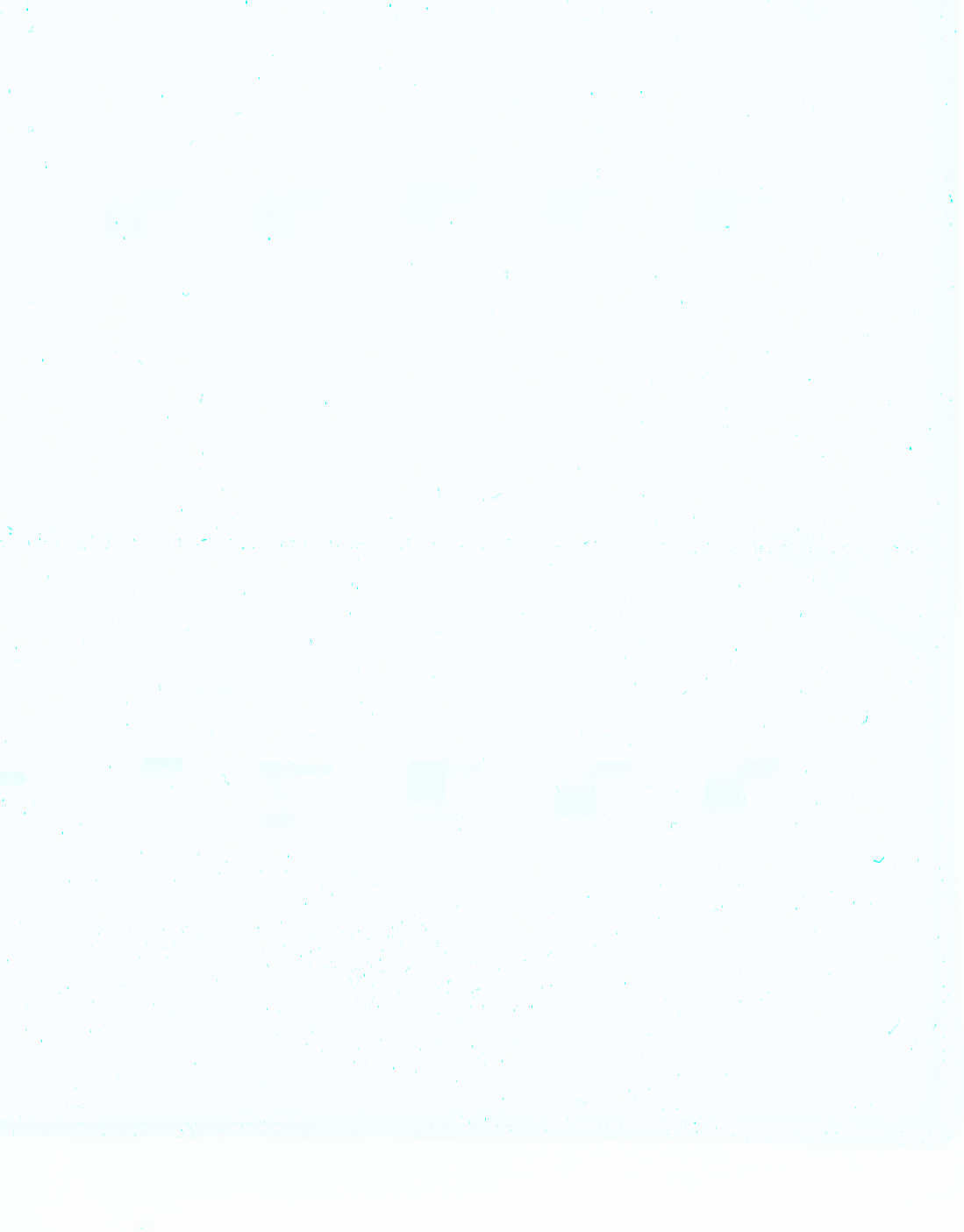

Supplement: Figure 1—figure supplement 1—source data 2. [file elife-67236-fig1-figsupp1-data2.zip › Figure 1-figure supplement 1B-source data 2.tif]

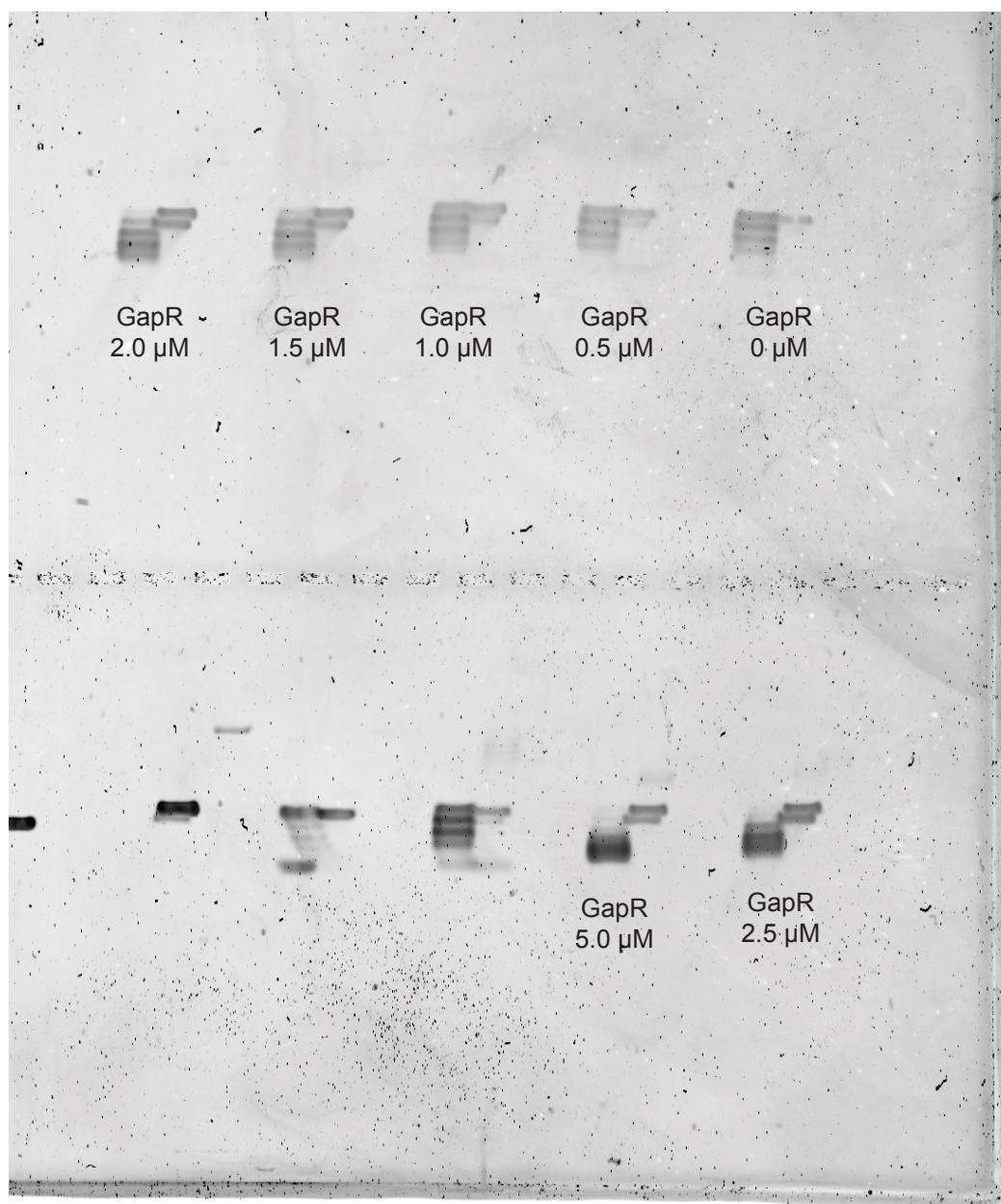

Supplement: Figure 1—figure supplement 1—source data 2. [file elife-67236-fig1-figsupp1-data2.zip › Figure 1-figure supplement 1B-source data 2_labeled.pdf]

☐ relaxed

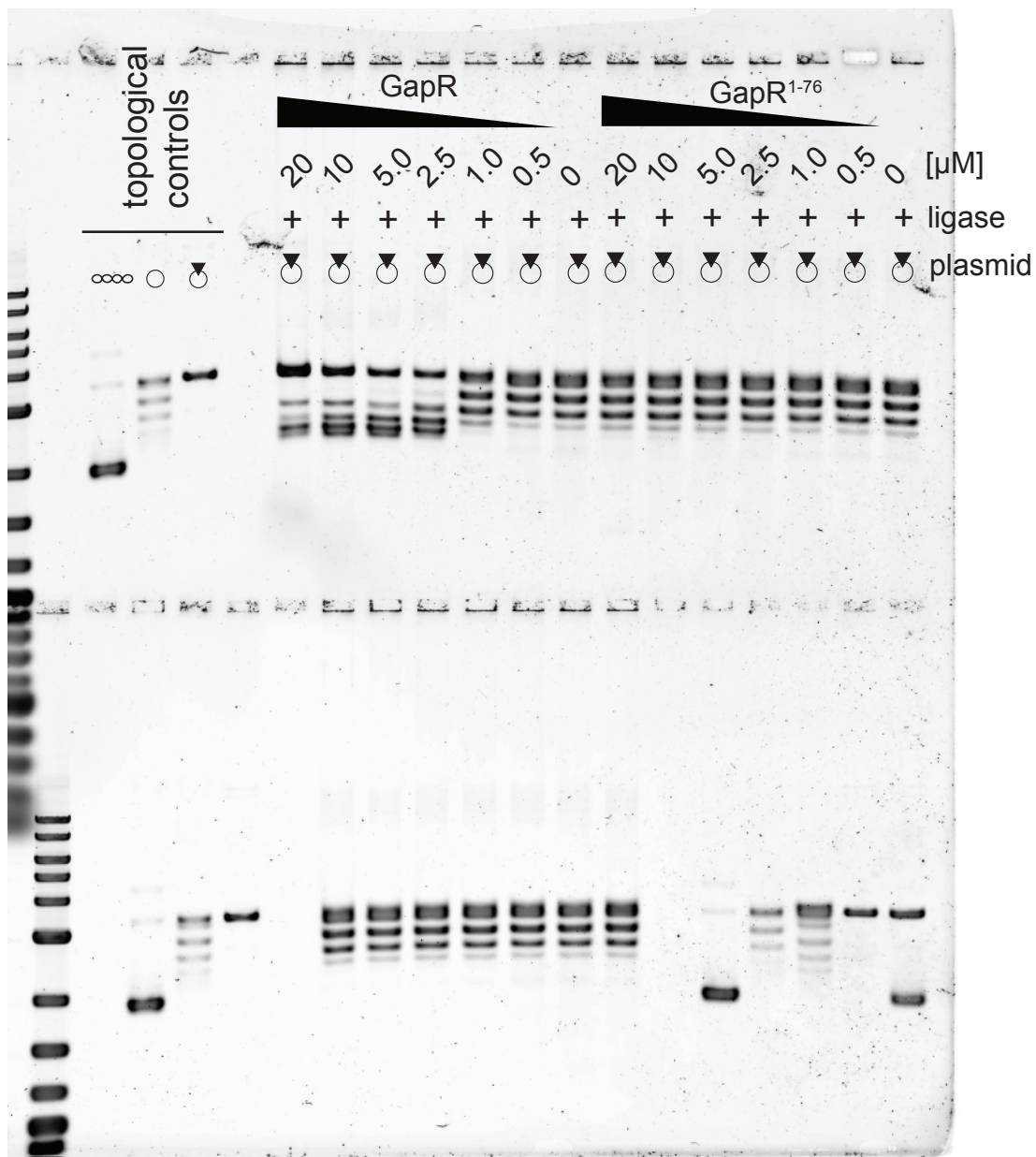

Supplement: Figure 3—source data 1. [file elife-67236-fig3-data1.zip › Figure 3A-source data 1_labeled.pdf]

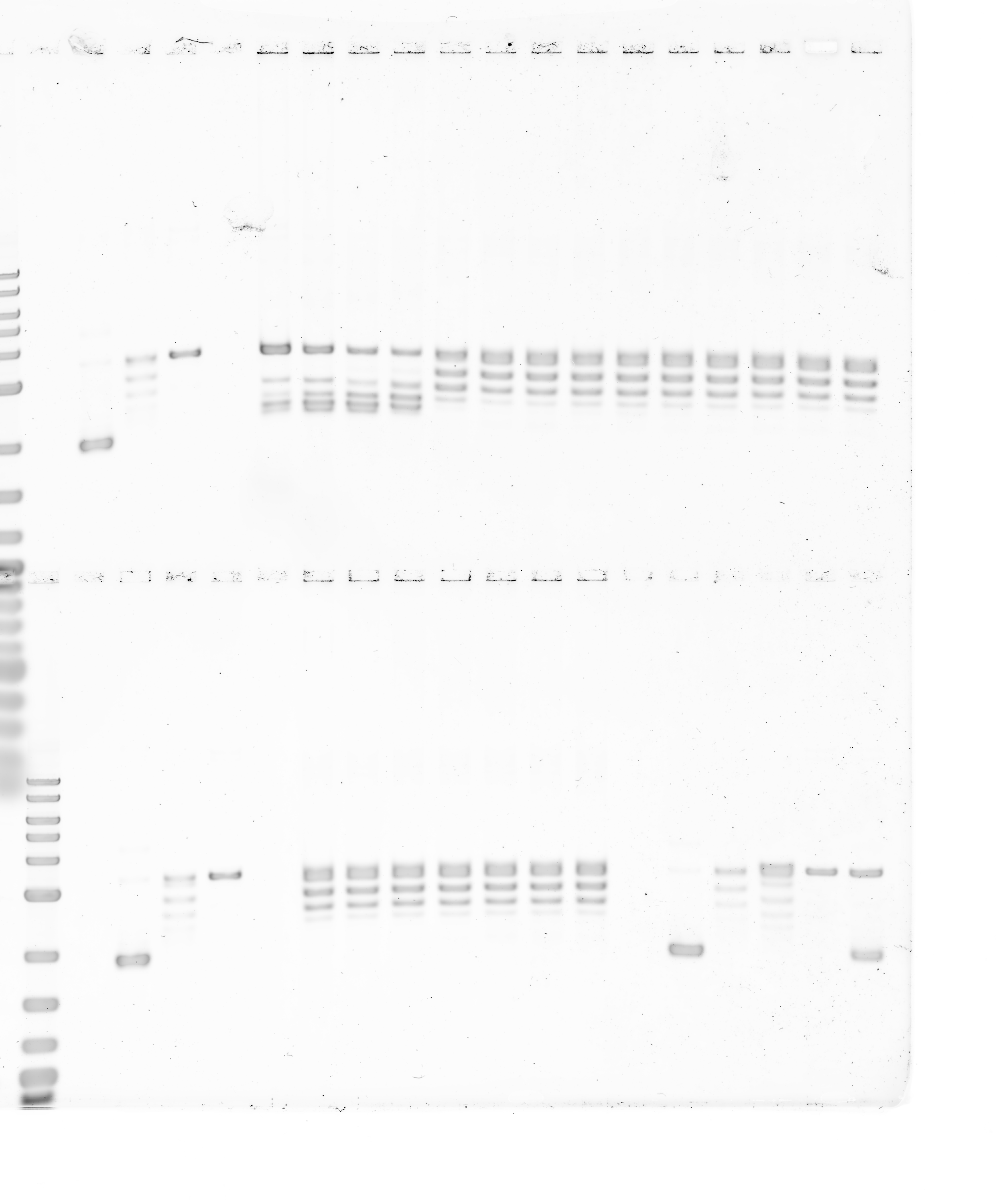

Supplement: Figure 3—source data 1. [file elife-67236-fig3-data1.zip › Figure 3A-source data 1.tif]

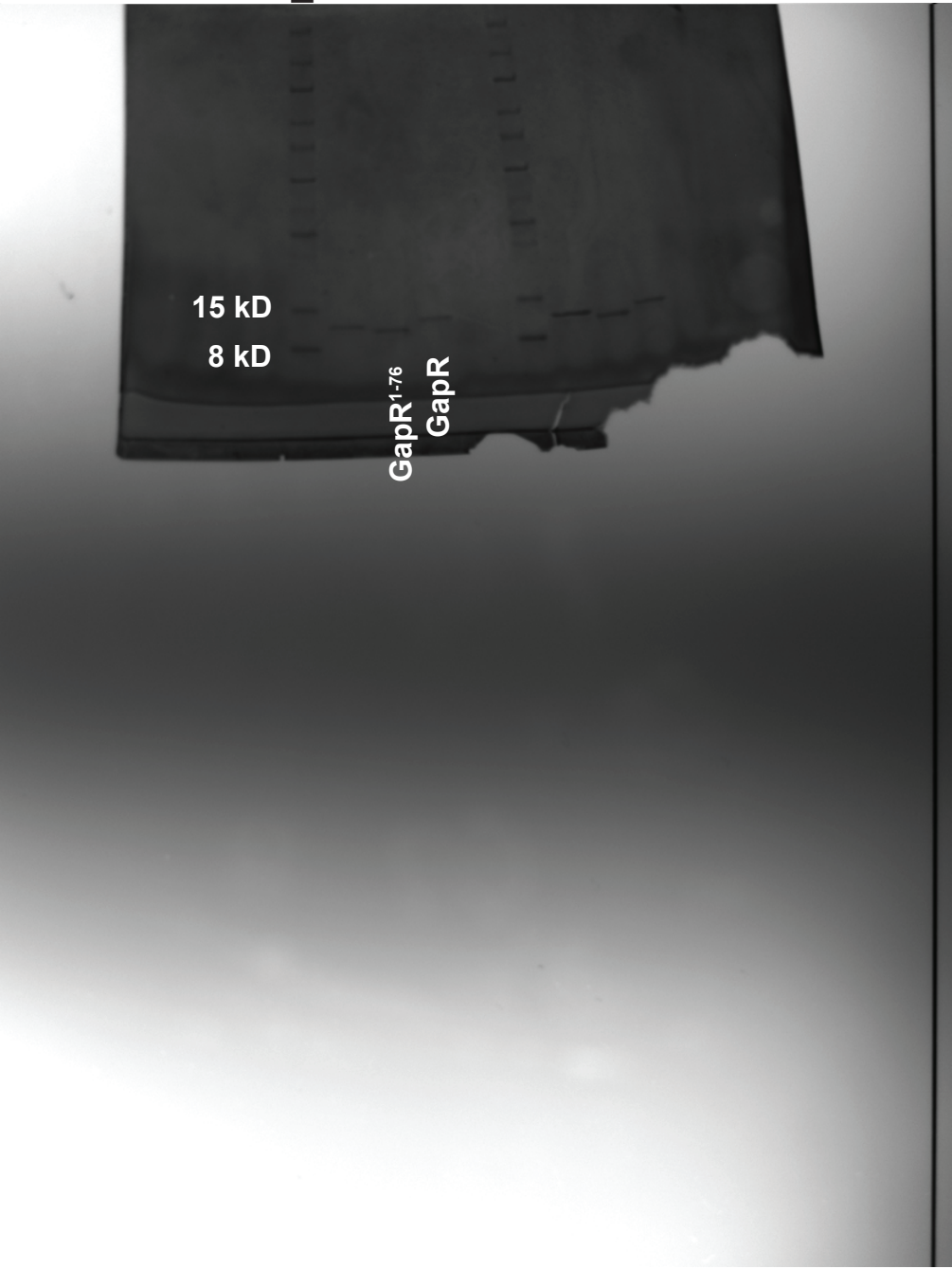

protein ladder

Supplement: Figure 3—figure supplement 1—source data 1. [file elife-67236-fig3-figsupp1-data1.zip › Figure 3-figure supplement 1A-source data 1_labeled.pdf]

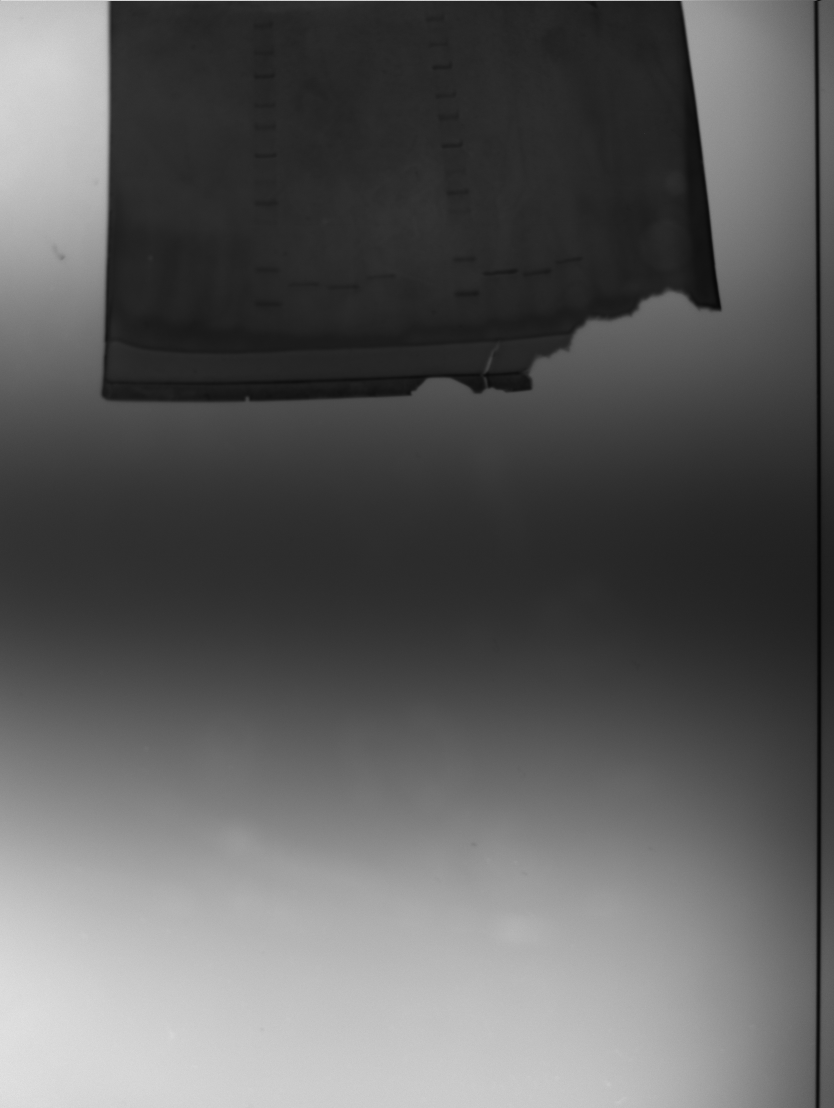

Supplement: Figure 3—figure supplement 1—source data 1. [file elife-67236-fig3-figsupp1-data1.zip › Figure 3-figure supplement 1A-source data 1.tif]

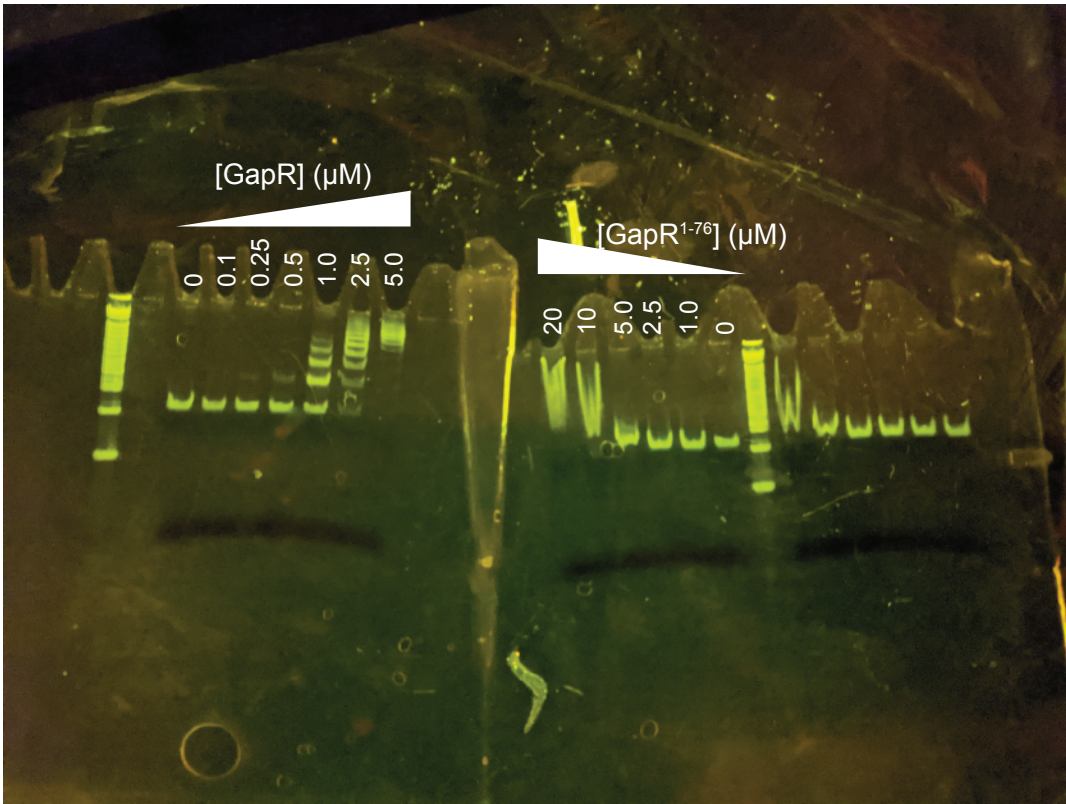

Supplement: Figure 3—figure supplement 1—source data 2. [file elife-67236-fig3-figsupp1-data2.zip › Figure 3-figure supplement 1B-source data 2_labeled.pdf]

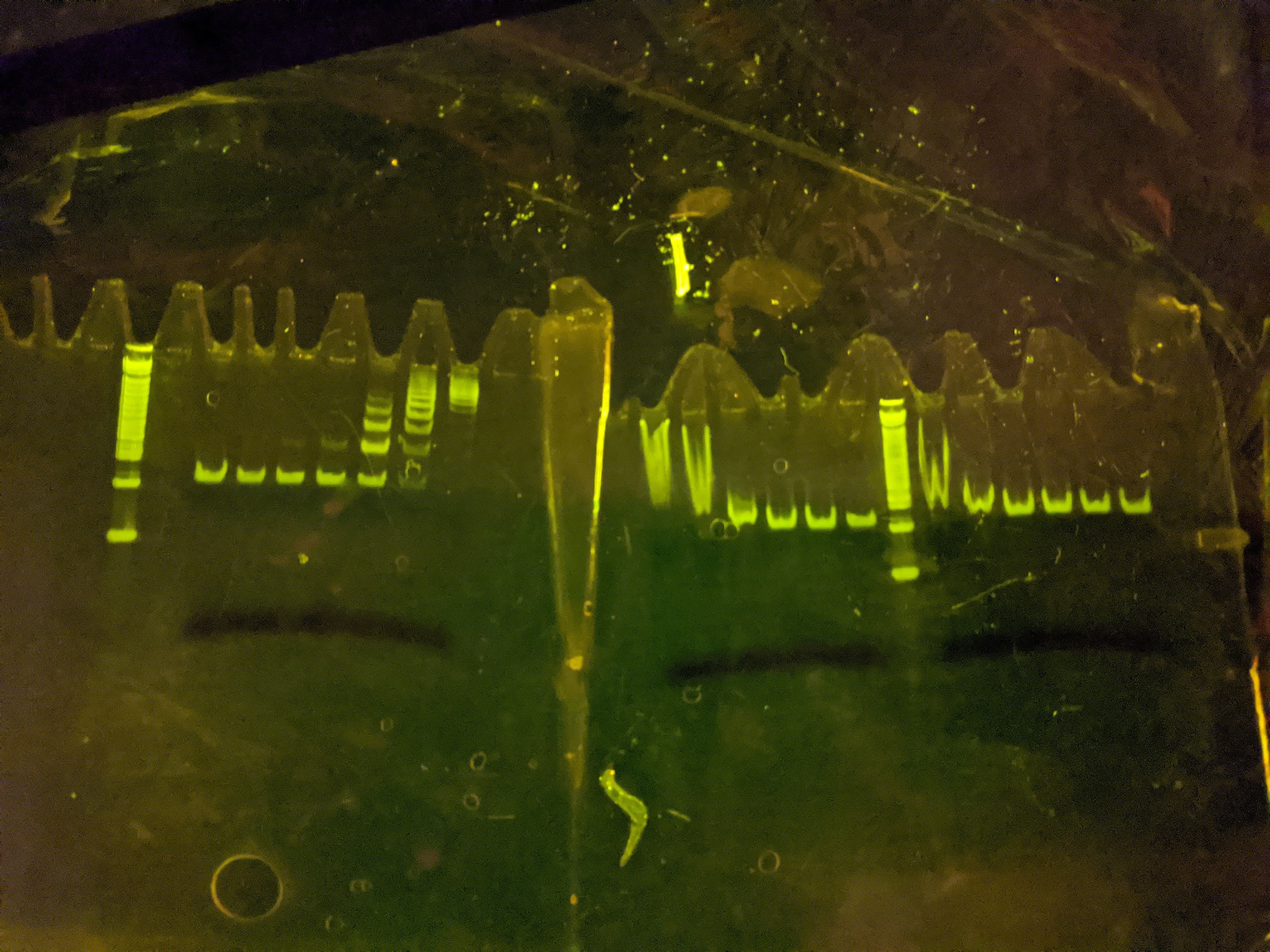

Supplement: Figure 3—figure supplement 1—source data 2. [file elife-67236-fig3-figsupp1-data2.zip › Figure 3-figure supplement 1B-source data 2.jpg]

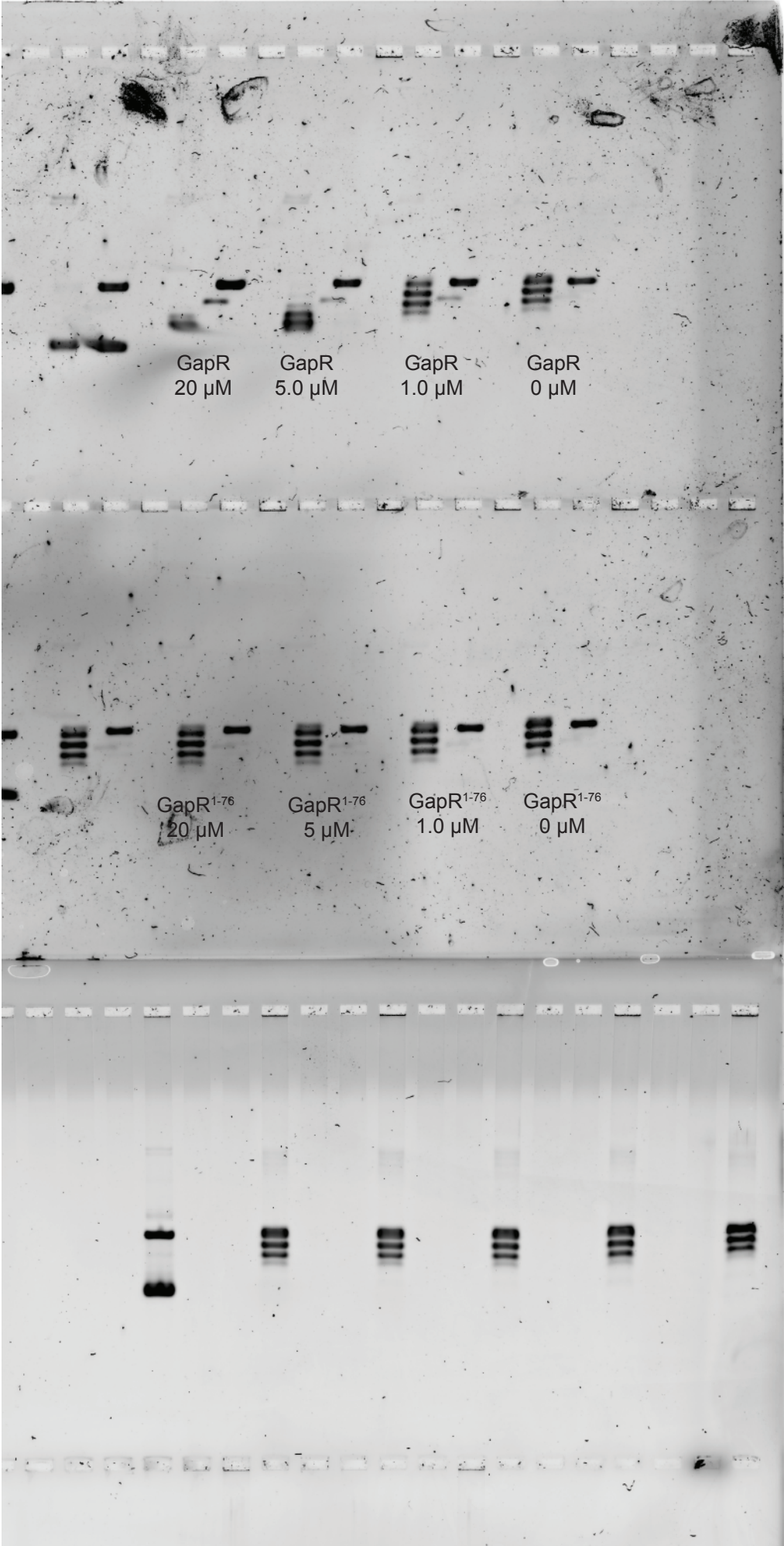

Supplement: Figure 3—figure supplement 1—source data 3. [file elife-67236-fig3-figsupp1-data3.zip › Figure 3-figure supplement 1C-source data 3-labeled.pdf]

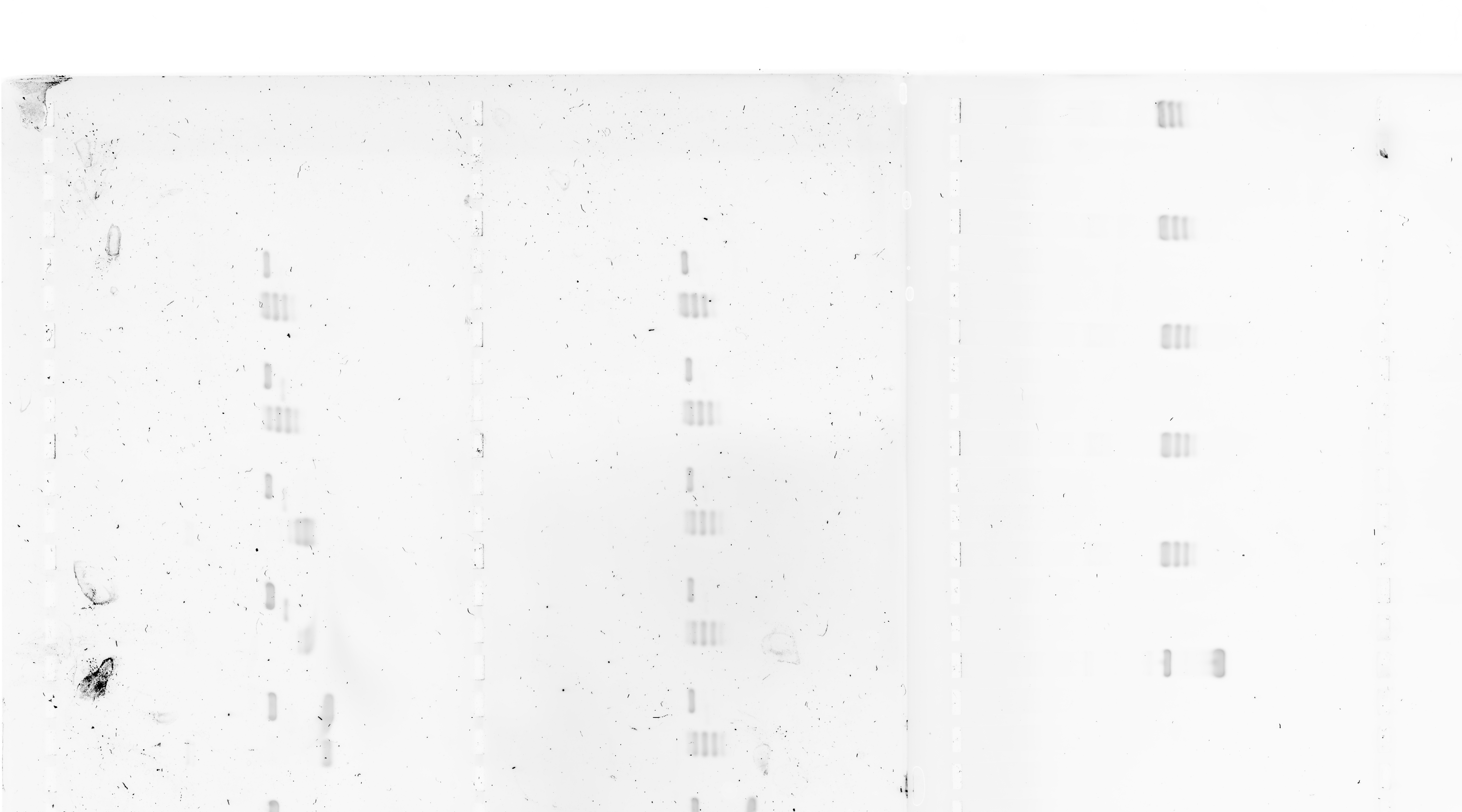

Supplement: Figure 3—figure supplement 1—source data 3. [file elife-67236-fig3-figsupp1-data3.zip › Figure 3-figure supplement 1C-source data 3.tif]
